# The European 2030 climate and energy package: do domestic strategy adaptations precede EU policy change?

**DOI:** 10.1007/s11077-022-09447-5

**Published:** 2022-02-05

**Authors:** Lana Ollier, Florence Metz, Alejandro Nuñez-Jimenez, Leonhard Späth, Johan Lilliestam

**Affiliations:** 1 Institute for Advanced Sustainability Studies (IASS), Berliner Strasse 130, 14467 Potsdam, Germany; 2 University of Twente, Enschede, Netherlands; 3 Department of Environmental Systems Science, ETH Zurich, Zurich, Switzerland; 4 Belfer Center for Science and International Affairs, Harvard University, Cambridge, USA; 5 Faculty of Economics and Social Sciences, University of Potsdam, Potsdam, Germany; 6 Group for Sustainability and Technology, ETH Zurich, Zurich, Switzerland

**Keywords:** Climate and energy policy, Policy strategy, European Union, Decarbonization, Renewable energy

## Abstract

The European Union’s 2030 climate and energy package introduced fundamental changes compared to its 2020 predecessor. These changes included a stronger focus on the internal market and an increased emphasis on technology-neutral decarbonization while simultaneously de-emphasizing the renewables target. This article investigates whether changes in domestic policy strategies of leading member states in European climate policy preceded the observed changes in EU policy. Disaggregating strategic change into changes in different elements (goals, objectives, instrumental logic), allows us to go beyond analyzing the relative prioritization of different goals, and to analyze how policy requirements for reaching those goals were dynamically redefined over time. To this end, we introduce a new method, which based on insights from social network analysis, enables us to systematically trace those strategic chances. We find that shifts in national strategies of the investigated member states preceded the shift in EU policy. In particular, countries reframed their understanding of supply security, and pushed for the internal electricity market also as a security measure to balance fluctuating renewables. Hence, the increasing focus on markets and market integration in the European 2030 package echoed the increasingly central role of the internal market for electricity supply security in national strategies. These findings also highlight that countries dynamically redefined their goals relative to the different phases of the energy transition.

## Introduction

In 2019, the European Union passed a series of laws as part of the 2030 climate and energy package, which includes important changes from its 2020 predecessor (EC, 2009a, 2009b, 2009c, 2012). First, whereas the 2020 package held binding domestic targets for renewables (EC, 2009b), the 2030 package does not. Instead, the 2030 three-pillar climate and energy targets for 2030 operate, with the exception of targets for non-ETS sectors,^1^ at the Union level and aspire to 40% greenhouse gas emission reduction (EC, 2018d), 32% increase in renewables (EC, 2018b), and 32.5% improvement in energy efficiency (EC, 2018c).^2^ In addition to a weaker legal status for renewables, the 2030 package focuses on emissions trading as the main instrument for decarbonization instead of support policies for renewables (EC, 2018a; Fitch-Roy et al., 2019). Second, the 2030 package more strongly emphasizes the role of the internal market by reinforcing institutional and technical guidelines and harmonizing policy support schemes for renewables to reduce market distortions (EC, 2018e).

Already during the negotiations of the 2020 climate and energy package (in 2007–2009), both legally binding renewables targets and the role of the internal market were controversially debated (Lauber & Schenner, 2011; Tews, 2015). On the one side, renewables support-focused actors advocated for ambitious legally binding renewables targets to require member states to accelerate the deployment of renewable energy such as wind, solar, hydro and biomass power, and solar or biomass heat. On the other side, supporters of technology-neutral decarbonization promoted a single, legally binding greenhouse gas (GHG) reduction target for the entire European Union. This position favored a strong emissions trading scheme and supported reconfiguring the existing system with low- or zero-carbon technologies (e.g., switch from coal to gas or nuclear power) and sequestration technologies (e.g., carbon capture and storage) (Gullberg, 2015; HM Government, 2014). The internal market was similarly contentious. Member states had varying concerns regarding supply security (Fischer, 2017b; Szulecki, 2016), and regarded the Commission’s push for harmonizing renewables’ support schemes to limit distortions of the internal market through different lenses. Member states using renewables as a “vehicle for regional development and job creation” strongly opposed harmonizing support schemes (Fitch-Roy et al., 2019; Strunz et al., 2019). Eventually, member states that focused on supporting renewables prevailed in the negotiations, and the 2020 package established binding national renewable energy and climate targets.

The 2030 package takes the opposite direction from its predecessor. Today, only the emission reduction target (and only the non-ETS part of it) is a legally binding target at the member state level (EC, 2018d). For renewables, member states are encouraged to take action within the internal market through increasingly harmonized support schemes, especially auctions, but they do not have binding national renewables targets (EC, 2018b). These differences between the 2020 and 2030 climate and energy packages suggest important changes in the policy positions of relevant actors during the negotiations leading up to the policy packages. Previous scholarship has explored changes in the 2030 climate and energy package from the perspective of non-state actors on the agenda-setting process (Fitch-Roy et al., 2019) and the increasing influence of Central and Eastern European countries (CEEC) (Braun, 2019; Ćetković & Buzogány, 2019; Skjærseth, 2016, 2018). However, there has not been a systematic analysis of changes in the policy strategies of the leading member states for EU climate and energy policy, such as Germany, France, Spain, Italy, the UK, and Sweden. These member states were highly influential during the negotiations of the 2020 energy package and their positions strongly affected the eventual content of the package adopted in 2009 (Lauber & Schenner, 2011). This study aims to fill this gap by addressing the question of whether and how the 2030 climate and energy package, adopted in 2019, was preceded by domestic changes in the policy strategies of EU climate policy-leading countries. Here we take a high-level view on the packages, refraining from going too far into detail for any of the acts combined in the packages (Table 1).

**Table 1:** Main differences between the 2020 and the 2030 climate and energy package

2020 Climate and Energy Package	2030 Climate and Energy Package
Binding, national renewable energy and climate targets Far-reaching member state autonomy over National support schemes	Binding national emission reduction targets Binding EU-level renewable energy targets Increasingly harmonized support schemes Increasing focus on the internal market

This question addresses a central conundrum within the Europeanization literature, where we find two dominant views on the forces that shape European integration. Intergovernmentalists apply a state-centric vision to the EU, where member states maintain the ultimate decision-making power (Moravcsik, 1993). Bargaining is therefore central to EU policymaking, and policy solutions are based on the least common denominator. Multi-level governance offers a different perspective, however, where member states—although they remain important actors—share decision-making powers with EU-level actors and institutions (Hooghe et al., 2001). Following this line of argument, states are exposed to policies that do not necessarily align with their own national preferences referred to as “misfit” between European policies or institutions and national preferences in the respective literature (Börzel, 2000, Börzel and Risse, 2003). Our case allows us to test whether EU institutions managed to prevail against domestic interests despite a misfit between national an EU interests, or whether the observed EU-level policy change resulted instead from changing national interests.

To address our research question, we present a method that goes beyond analyzing changes in the prioritization of different energy policy goals and allows us to consider the dynamic (re)interpretation of climate and energy policy goals. Building on central concepts used in studying social networks, we analyze how countries’ governments match their goals with specific policy requirements over time. For this purpose, we prepare a dataset manually coding 2237 pages of domestic policy strategies from six countries.

This method offers a different perspective on policy change and enables us to make empirical contributions to the existing literature. First, we explore the policy change in the European climate and energy packages from the perspective of climate-politically influential member states. Second, we show how EU policy change is linked to a reconfiguration of domestic strategic goals. While analyses of the relative importance of different energy policy goals exist for individual states (e.g., Germany (Schmidt et al., 2019)), we add a comparative analysis to this literature. This not only helps us to gain a more complete picture of the drivers of energy and climate policy in different states, but also provides us with insight into the ways in which domestic goals shape the outcome of inter-state negotiations.

In the following sections, we introduce our conceptual framework, define hypotheses for changes in countries’ policy strategies based on the transformation of the electricity system between the 2020 and 2030 climate and energy package adoption, and describe our method and data. Our results in Sect. 5 are structured in two parts: the first investigates changes in countries’ strategies over time by analyzing changes in the relative importance of individual goals, objectives, and the dominant instrumental logic; the second part analyzes how these goals and objectives are linked to each other as part of each country’s unique argumentative logic. We then discuss our findings in the context of ongoing system changes as part of the energy transition, before concluding with an outlook on policy implications for the implementation of the 2030 climate and energy policy.

## Conceptual framework

In this study, we test whether the changes in the 2030 climate and energy package were preceded by shifts in leading climate-policy state policy strategies. We look at strategic change as alterations in a country’s unique combination of ends and means related to their climate and energy policy.

**Fig. 1:**
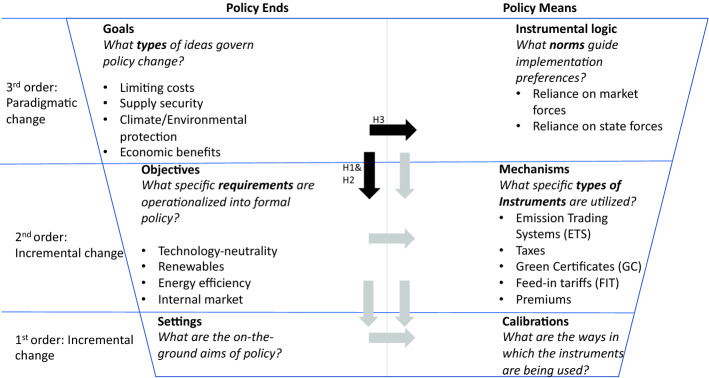
Taxonomy of policy design elements in energy policy based on Cashore and Howlett (illustration based on (Haelg et al., 2020)). In the bullet points, we apply the taxonomy of policy design elements to the field of climate and energy policy, where the central elements identified served as coding categories. The grayed-out design elements are not investigated in this study. The arrows indicate the direction of influence; here we assume that higher-level policy ends/means influence lower ones (vertical arrows), but also that policy ends influence policy means (horizontal arrows). We test the dynamic formulation of policy strategies for both of these dynamics in three hypotheses (H1, H2, and H3)

We apply Cashore and Howlett’s taxonomy of variable policy elements (Cashore & Howlett, 2007). Their taxonomy highlights that policy is “a complex regime of ends and means,” where they distinguish between six different “levels or orders” that may undergo change: goals, objectives, settings, instrumental logic, mechanisms, and calibrations (Fig. 1). These categories align with Hall’s (1993) conception of “incremental” (first- and second-order policy change) versus “radical” (third-order policy change) (Fig. 1). Starting at the bottom of Fig. 1, in Hall’s (1993) terminology, changes in settings and calibrations constitute the smallest possible policy alterations and are termed “first-order change.” In the middle are “second-order changes” that occur in objectives and mechanisms. At the top are third-order changes, i.e., major policy changes in goals and instrumental logics (Hall, 1993). We employ this distinction to gain a more nuanced understanding of domestic policy developments and to assess the degree and type of strategic change. Oftentimes, scholars focus on the influence of higher- on lower-level policy ends and higher-level on lower-level policy means (Haelg et al., 2020; Hall, 1993) (vertical arrows in Fig. 1). However, the links between design elements at the same level (horizontal arrows in Fig. 1) have also been recognized (Howlett, 2009). How and whether there is “diagonal influence”—for example, linking higher-level ends to lower-level means—should be the focus of further research, but we do not consider such links here. Focusing on the vertical and horizontal directions of influence, the arrows in Fig. 1 show all possible influence dynamics that can be tested for coherence. Here, we focus on the influence of goals on objectives and instrumental logic (the black arrows in Fig. 1), because our research question concerns strategic policy reorientation, and not the details of instruments. This focus does not only address a gap in the climate and energy policy literature, which typically focuses on countries’ choices of mechanisms (Ćetković & Buzogány, 2016; Lauber & Schenner, 2011, Meckling et al. 2015). As renewable electricity support mechanisms that have been strongly harmonized in the 2014 State Aid Guidelines (EC, 2014), we expect only little variation in Mechanisms, Settings, and Calibrations in TP 2.

From an institutionalist perspective, design elements are oftentimes treated as matching sets, where prefixed goals determine objectives, which in turn determine mechanisms (Ćetković & Buzogány, 2016; Hager, 2015; Stefes, 2014). While previous studies observe changing policy objectives alongside stable policy goals (Schmidt et al., 2019), it is not clear how the two elements are dynamically linked as part of establishing a causal chain. We address this gap by treating element combinations as flexible and open to interpretation: we assume that all combinations of goals, objectives, and instrumental logic are possible and that political interpretations of causal chains may change over time. Concretely, actors may not only re-prioritize goals, but also redefine them by linking goals to new or different objectives and instrumental logics over time. We find an example of this in the Juncker administration, which successfully linked the objective of the internal electricity market to climate and environmental policy, as opposed to the previous link to supply security (Fischer, 2017b). In this study, we refer to such redefinition as changes in the argumentative logic.

Every policy field consists of a variety of different competing policy goals (Howlett & Rayner, 2007; Kern & Howlett, 2009). Prioritization, based on normative decisions, between these potentially conflicting goals is thus a necessary outcome of the policy-making process. Previous scholarship identifies three key policy goals for the energy sector: (i) limiting costs^3^ (ii) securing the supply of energy, and (iii) reducing the environmental- and particularly climate burden (Helm, 2002, 2005; Hughes & Lipscy, 2013; WEC, 2016). Balancing these three overarching goals, which are generally referred to as the “energy policy triangle” or “trilemma,” shapes energy policy (Costa-Campi et al., 2017; Quitzow, 2015; Winzer, 2012). Schmidt et al. (2019) added “increased competitiveness of domestic energy technology industry” as a fourth policy goal to the trilemma.

The policy design element “objectives” describes the specific requirements that are operationalized into formal policy (Cashore & Howlett, 2007). Previous research has shown that states pursue different objectives regarding their technological approach to decarbonization (Komor & Bazilian, 2005; Ollier et al., 2020; Patt et al., 2019). For the energy sector, three broad strategies for decarbonization are available: (i) reconfiguration of the existing system and a transition to lower-carbon options (e.g., modernizing coal power plants, transitioning from coal to gas power, nuclear energy, or carbon capture and storage) in a technology-neutral approach, (ii) deployment of renewables, and (iii) the reduction of demand, and therefore consumption, through energy-efficiency measures (IPCC, 2018). Here we add a fourth objective that continues to be of unequal interest to the EU’s member states: the establishment of the internal electricity market (Fischer, 2017a; Szulecki et al., 2016).

Goals and objectives are implemented in adherence to an “instrumental logic”—guiding norms in the implementation process. In this study, we focus on reliance on “market forces” versus “state forces” in implementing climate and energy policy. “State forces” are here identified as positive mentioning of regulation or of the state taking action (e.g., direct investment), or strongly guiding other actors implementing actions. “Market forces,” in contrast, are identified through the positive mentioning of the need to rely on markets and/or (private) actors playing a key role in implementing actions, but also positive mentioning of liberalization and deregulation. Both instrumental logics are well researched in the context of climate and energy policy (Schaffrin et al., 2014, Ćetković & Buzogány, 2016). This research highlights how, based on states’ preferred instrumental logic and their unique features in delivering social and economic benefits, concrete mechanisms (or instrument types) are implemented.

## Policy goals and objectives in a transitioning system

Fueled by the growing deployment of renewables, the 2030 climate and energy package marks a new phase in the energy transition. Renewables started out as niche innovations with high costs and far-reaching requirements for support, but they have progressively outgrown this niche and now compete with incumbent fossil fuel and nuclear power (Geels, 2014; Markard, 2018). Nonetheless, the transition does not stop there. An increasing prevalence of intermitted renewables presents a challenge for today’s energy system, which is further challenged by the integration of other sectors in the energy transition (Markard, 2018; Sinsel et al., 2020). As such, this second phase of the transition has new infrastructure and institutional needs, amplified by the increasing interaction of many parallel transitions across multiple sectors.

Previous research shows that changes in the socio-technical system shape the choice of policy instruments and the configuration of policy mixes, whereby successful instrumentation occurs “relative to the phase of the transition” (Edmondson et al., 2019; Pahle et al., 2018; Schmidt & Sewerin, 2017; Schmidt et al., 2019). Those findings suggest that goals, objectives, and the dominant instrumental logic change according to the phase of the transition. The new transition phase is therefore likely to affect member states’ strategic goals and objectives. They will face new challenges and opportunities, which could result in paradigmatic changes in national goals, objectives, and their dominant instrumental logic, which in turn could then influence synergies and coalitions between countries. We therefore expect system change to be a motor behind (a) changes in the prioritization of different policy design elements, and (b) changes in countries’ argumentative logic where different goals and objectives are combined. Here it is important to acknowledge that the countries in our sample are not all in the same phase of the energy transition, with different shares of renewables and different renewable energy growth rates (see Appendix A2). Here, we do not investigate how the national transition phase affects policy choices, but rather how countries go about their national decarbonization process and position themselves in the EU transition process, and encourage further research on how the challenges and opportunities of the different transition phases shape domestic strategies.

Against this backdrop, we formulate three hypotheses to test whether countries show common trends in redefining their policy strategies in a way that would precede and help explain the observed policy changes in the 2030 climate and energy package. First, as renewables gain market share, the increasing amount of variable supply presents a challenge for countries’ current power systems. The share of variable supply varies between countries depending on their renewables’ growth rate and the resulting relative importance of other sources, such as baseload nuclear power or flexible gas power. In order to ensure energy security, a system with high penetration of renewables needs increasing system flexibility (Tagliapietra et al., 2019). Flexibility in a high-renewables system can be provided locally through storage systems and demand-side management, or by increasing the size of the system (distributed vs. centralized) (Tröndle et al., 2020). The latter relies on strong integration of the EU’s internal market and smoothing of supply fluctuations through the power grid (Thonig et al., 2020). While the push for the internal market has historically come predominantly from the European level (EC, 1999, 2014; ENTSO-E, 2018), increasing flexibility requirements over time may lead member states to favor the internal market as a means to deal with higher shares of variable supply and to maintain electricity security. Therefore, we expect that the policy objective of “*internal markets*” and the goal of “*supply security*” become increasingly central and linked in member states’ policy strategies. In this case, domestic changes precede the increased focus on the internal market in the 2030 package. We differentiate here between two time periods: the last published national strategy prior to adopting the 2020 climate and energy package (TP1), and the last strategy prior to adopting the 2030 climate and energy package (TP2).

### Hypothesis 1

*As the share of variable renewable energy supply increased between TP1 and TP2, supply security concerns grew and increased the relative importance of the internal energy market in domestic policy strategies of climate policy-leading countries.*

### Hypothesis 1.1

*Between TP1 and TP2, supply security and internal markets became increasingly central in domestic energy policy strategies.*

### Hypothesis 1.2

*Between TP1 and TP2, supply security and internal markets became increasingly linked in domestic energy policy strategies in forming a new argumentative logic.*

Second, in 2009, the EU adopted legally binding renewable energy targets for the first time. In that phase of the transition, renewables were still costly and member states adopted renewables’ support instruments to pull renewables into the market and to achieve the deployment targets. Here, feed-in tariffs were deemed particularly effective in fostering technological development (Jacobs, 2012). However, from the start, the European Commission and the power industry (Scott, 2018), along with energy and climate economists (Böhringer et al., 2009), criticized the EU’s three-target approach—including a designated renewables target—and instead argued for a single, technology-neutral target backed by carbon pricing. They argued that a single target approach would trigger least-cost decarbonization by promoting market-ready low-carbon alternatives instead of less mature renewable technologies. Advocates for renewables, prominently the German government, which at the same time pursued a nuclear phase-out, opposed such a single-target approach to decarbonization (Geden & Fischer, 2014). Since then, however, states increasingly sought to control the increasing costs of renewables support schemes (Leiren & Reimer, 2018). On the one hand, costly support schemes, especially high feed-in tariffs, were becoming increasingly unpopular during the economic crisis (Gürtler et al., 2019; Leiren & Reimer, 2018). On the other hand, the failure of the incumbent utilities in some countries, notably Germany, to invest in renewables, had brought them close to bankruptcy (Leiren & Reimer, 2018; Stenzel & Frenzel, 2008). As a consequence, advocacy in favor of a strong technology-neutral emissions target (and a relatively weaker renewables target) may have fallen on fertile ground in the 2030 climate and energy package (Fitch-Roy et al., 2019). We therefore expect cost concerns (“*limiting costs”* in Fig. 1) to a) become an increasingly central goal, and b) to be linked to the objective of “*technology-neutrality*” in forming a new argumentative logic.

### Hypothesis 2

*As the cost of renewables’ support schemes increased, cost concerns grew and increased the relative importance of technology-neutral decarbonization in domestic energy policy strategies between TP1 and TP2*.

### Hypothesis 2.1

*Between TP1 and TP2, limiting costs and technology-neutral decarbonization became increasingly central in domestic energy policy strategies.*

### Hypothesis 2.2

*Between TP1 and TP2, limiting costs and technology-neutral decarbonization became increasingly linked in domestic energy policy strategies in forming a new argumentative logic.*

Third, the goal of “limiting costs” may not have only influenced member states’ objectives, but also have affected their preferred instrumental logic. As already mentioned, with growing criticism from public and utilities, in particular in the aftermath of the 2009 European debt crisis, states increasingly found themselves in a situation of needing to control the costs of renewables’ support schemes, also to comply with rescue bond criteria (Leiren & Reimer, 2018, Gürtler et al., 2019). Previous research shows that an instrumental logic building on the reliance on market forces is generally associated with cost effectiveness and an expectation of lower costs through market competition (Meckling, 2019, Dolšak and Sampson, 2012). In addition to driving member states toward more technology-neutral support schemes, we hypothesize that they more strongly emphasize an instrumental logic relying on market forces to limit costs through competition. We therefore expect cost concerns (“limiting costs” in Fig. 1) to a) become an increasingly central goal, and b) be linked to the objective of “reliance on market forces” in forming a new argumentative logic.

### Hypothesis 3

*As the cost of renewables’ support schemes increased, cost concerns grew, thereby increasing the relative importance of a ‘reliance of market forces’ in domestic energy policy strategies between TP1 and TP2.*

### Hypothesis 3.1

*Between TP1 and TP2, limiting costs and reliance of market forces became increasingly central in domestic energy policy strategies.*

### Hypothesis 3.2

*Between TP1 and TP2, limiting costs and reliance of market forces became increasingly linked in domestic energy policy strategies in forming a new argumentative logic.*

We test H1, H2, and H3 by comparing the ways in which supply security and growing cost concerns (as goals)—in addition to the internal market and technology neutrality (as objectives) together with a state-driven versus market-driven logic (as instrumental logics)—are embedded in countries’ energy policy strategies in TP1 and TP2. To reduce the risk of confirmation bias, we test whether countries’ strategies have shifted along these specific goals, objectives, and instrumental logics, and whether they have become more central compared to other goals, objectives, and the alternative instrumental logic (cp. Figure 1). First, we analyze whether the five strategic goals, objectives, and instrumental logic in question— “internal market,” “supply security,” “limiting costs,” (with its sub-categories, see Fig. 1), “technology neutrality” and “reliance on market forces”—gain centrality in member states’ strategies (H1.1/H2.1/H3.1). Second, we test whether these goals and objectives become more linked, thereby forming a new argumentative logic (H1.2/H2.2/H3.2). If our hypotheses are supported, member states argue in favor of internal markets in order to improve supply security (H1), in favor of technology neutrality (H2) and reliance on market forces (H3) to limit costs (H2).

## Data and methods

### Data & case selection

We select the member states in our sample in order to assess whether the most influential EU climate policy-leading countries have repositioned themselves in the field of climate and energy policy. The term “leader” has multiple definitions (Andresen & Agrawala, 2002), and here we focus on political leadership as defined by the countries themselves in establishing the *Green Growth Group*. This group consists of 14 similar-minded governments^4^ that are generally considered a central driving force behind ambitious EU climate policy and which are jointly pushing for action to limit global warming at 1.5° (GGG 2018, 2014). Informed by previous analysis on conflict lines within this group (Lauber & Schenner, 2011, Wettestad et al., 2012), we select a sample of six countries from the Green Growth Group: Germany, France, the UK, Spain, Italy, and Sweden. Previous literature shows that the conflict line between these states in the negotiations of the 2020 climate and energy package ran between states pushing for a national, technology-specific, and price-based renewables support (esp. FITs) and showing no agreement with further market integration on the one side. Meanwhile, on the other side, states were pushing for technology-neutral support using more market-based approaches within a strengthened internal market (Boasson & Wettestad, 2014; Lauber & Schenner, 2011; Schmidt et al., 2019). In the 2020 package negotiations, Spain and Germany could be found on one side, and the UK, the Commission, and Sweden on the other (Elliott, 2005; Hildingsson et al., 2012; Lauber & Schenner, 2011; Strunz et al., 2015). By adding France and Italy to our sample—countries that are known to have been decisive actors for the new targets in the 2030 package, (Darby, 2018; Simon, 2018; Vaughan, 2018)—we investigate how this conflict line developed to the negotiations of the 2030 climate and energy package. We collect data from the climate and energy strategies of these countries for two time periods (TPs): prior to adopting the 2020 climate and energy package (TP1) in 2009, and prior to adopting the 2030 climate and energy package (TP2) in 2019. We use the most recent strategy document as indicative for each county’s strategic position in the respective TP. The time perspectives of the implemented national strategies are not always identical to the one negotiated in the respective EU-level process. However, the existing strategy and overall policy approach is very likely to correspond to the country’s domestic position in these TPs, assuming that countries significantly altering their position would also adopt a new domestic strategy.

We collect data from executive policy strategies. Contrary to formal policy documents, these strategies define not only mechanisms, settings, and calibrations, but also define mid- and long-term goals and objectives for climate and energy policy. Irrespective of negotiation tactics, these documents allow us to capture governments’ “undiluted” domestic positions. Specifically, our sample includes policy strategies (all countries), sometimes complemented by White Papers (e.g., for the UK). We select final versions of strategy documents that hold at least one of the following keywords in their title: “Renewable energy/electricity,” “Energy/electricity,” “Energy Transition,” “Climate,” “Decarbonize/-ing/-ization,” “Carbon,” and “Greenhouse gas” (or equivalent terms in the national language). We exclude strategies exclusively referring to decarbonization of transport or heat, and focus exclusively on the supply of electricity, which has been the main area of discord between EU countries (Jacobs, 2012; Kitzing et al., 2012; Lauber & Schenner, 2011; Strunz et al., 2015). The document selection for each case is discussed between at least one coder and the lead author to ensure coherence. For a list of all coded documents, see Appendix A (Table 2).

All documents are read and manually coded employing category-based content analysis (Fig. 1 indicates coding categories). All countries’ strategies are coded in their respective national language. During the coding process, the coder examines each sentence individually and decides a) whether it expresses a strategic aim relevant to a policy design element in Fig. 1, and b) to which category in the coding scheme it belongs. Each element is coded every time it occurs throughout a document. Since our analysis aims to account for linkages between policy elements, one sentence could be coded as expressing several different design elements. The strategies are coded by four different researchers knowledgeable on EU and national energy policies in the countries they coded, and native speakers in the national language of these policies. To increase inter-coder reliability, all coders analyzed one common text individually and discussed and resolved differences in the outcome where they occurred. This process was repeated with different texts until coded samples showed a high level of consistency (around 80%). We collected 4876 statements from 12 strategic documents using the software Discourse Network Analyzer (Leifeld, 2016). For data-triangulation, coders keep a logbook in which they record qualitative insights on “policy ends and means” along with the main themes of each strategy.

### Data analysis

To test our hypotheses, we proceed in two analytical steps in which we analyze a) which goals, objectives and instrumental logic become more central in climate policy-leading countries’ policy strategies (H1.1/H2.1/H3.1), and b) whether climate policy-leading countries change the argumentative logics by which they link policy goals and objectives (H1.2/H2.2/H3.2). Beyond understanding which goals and means have become more important for this set of influential, climate policy-leading countries in the EU, this analysis allows us to understand how the interpretation of those goals changed between TP1 and TP2.

#### Relative element centrality in domestic strategies

In the first step, we analyze which goals, objectives, and instrumental logic are central in countries’ policy strategies in both TPs. To this end, we assume that elements gain centrality the more they are strategically linked to other policy elements to form a cohesive strategy. We use (node-) centrality (Scott, 2012) as the main measure of importance for policy design elements under the assumption that more central elements are deeper embedded in the overall strategy. With this method, we go beyond counting words and investigate countries’ unique argumentative logic. We consider elements to be linked if they are mentioned within the same sentence. We recognize that this is a very strict criterion, but it is systematically applicable across all countries, in addition to being replicable. We measure centrality as degree centrality in an undirected network, i.e., the number of times a goal or objective is linked to other policy design elements. Dividing this value by the total number of links made (for each country in each of the three categories of goals, objectives, and instrumental logic) gives us the relative centrality (represented as a percentage) of a design element vis-à-vis the other elements in that category. The use of relative centrality, as opposed to absolute centrality, facilitates cross-country comparisons, as the strategy documents differ in length. The focus of our analysis is on goals, objectives, and the dominant instrumental logic. While our focus is on goals, objectives, and the dominant instrumental logic, we also consider these elements to increase in centrality if they are linked to concrete mechanisms (see Fig. 1). In practice, this approach means that in our analysis of how elements are embedded in the overall strategy, we also consider, e.g., a goal to rise in centrality if the goal is linked to a mechanism.

To compare the centrality of different goals and objectives across countries and time, we display them in a heatmap, with darker shades indicating higher centrality. We divide the relative design element centrality based on the structure of the data into three groups: maximum centrality (> 40% of the links), medium centrality (20–40%), and minimum centrality (< 20%). Using these groups, we can also calculate a change rate of centrality as the total number of changes, irrespective of whether it is positive or negative, between TP1 and TP2, both across countries and categories (goals, objectives, and instrumental logic). We calculate the centrality change rate by recoding the groups as follows: no mentioning = 0 points, minimum (< 20%) = 1 point, medium (20–40%) = 2 points, maximum (> 40%) = 3 points. We record changes between TP1 and TP2 as absolute values: if an element is not mentioned at all in TP1 (0) and mentioned at a maximum level in TP2 (3), this element has a change rate of 3. We calculate the sum of these changes per country and category. By means of the centrality change rate, we identify common trends of strategic change between TP1 and TP2 across countries and categories. Finally, we triangulate our analysis with qualitative information about policy strategies from the logbooks kept by the coders as part of the coding process.

#### Argumentative logic in domestic strategies

In the second step, we concentrate on changes in the argumentative logic. We analyze which specific goals and objectives are tied together in what we here refer to as “link centrality” (in social network analysis usually “edge centrality”) (Scott, 2012). Analyzing the ways in which climate policy leader states in our sample tie specific goals and objectives together creates additional insight into their interpretation of individual policy issues and how this interpretation changes over time (Baumgartner & Mahoney, 2008; Jones et al., 2014).

Links can be additive, where, e.g., two goals (goal/goal) or two objectives (objective/objective) are linked as equally desirable; they can also be causally linked, where, e.g., an objective is linked to a goal (goal/objective) in defining a formal policy requirement. Our hypotheses relate to such causal links. We hypothesize that increasing costs and supply security concerns drive repositioning on technology-neutrality, the reliance on market forces, and the internal market, respectively. Therefore, we focus on investigating causal links (goal-objective and goal-instrumental logic) with the help of an affiliation matrix, which reflects the goal-objective and goal-instrumental logic combinations of each country in our sample. We then investigate cross-country trends in linking the same goals and objectives or instrumental logic between TP1 and TP2.

## Results

Our results show that between TP1 and TP2, climate policy-leading countries in the EU increasingly linked “*supply security*” to “*internal market,*” but that they did not place these elements more centrally—because “*supply security*” remained a highly central goal. Hence, this finding supports H1.2, while H1.1 is rejected. Our results therefore indicate that instead of finding increasing recognition in TP2, “*supply security*” became redefined and expanded to also include “*internal market*” as a central policy requirement.

We also show that although “*limiting costs*” gained centrality between TP1 and TP2, the same did not hold true for “*technology neutrality*”—and the two concepts were not at all linked in TP2. This finding therefore rejects both H1.2 and H2.2. Concretely, this result indicates that, while the element of “*limiting costs*” became increasingly prioritized, and countries were likely to find agreement around this goal, “*technology neutrality*” did not play a role in its implementation. The following results explore these findings in full detail.

### Design element centrality within the policy strategies of climate policy-leading countries

**Fig. 2:**
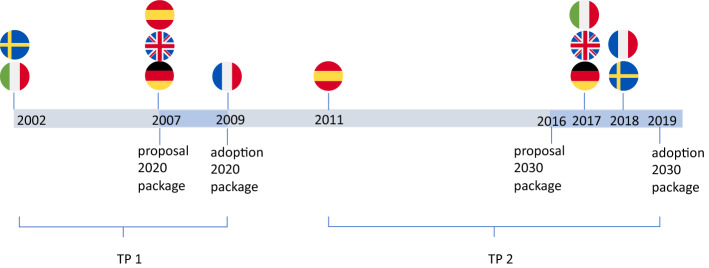
The two time periods investigated, and the time of publications of the national strategies used to identify the national positions. Negotiation periods are marked in blue between the initial Commission proposal and the adoption of the final package

Figure 2 shows the centrality of different design elements for the six countries in our sample and both TPs. On the X-axis, both TPs are displayed, grouped according to their category affiliation: goals, objectives, or instrumental logic. Elements with a higher degree centrality are marked by a darker shade. The absolute centrality change rate is indicated in the dark gray column/row. A higher change rate along the X-axis indicates a greater number of changes in countries’ central interest. A higher change rate along the Y-axis indicates a greater number of changes within the element indicated to the left.

While some states changed their preferences, we observe no common trend for EU climate policy-leading countries in increasingly prioritizing “*supply security*” in TP2 (see Fig. 2). Although the “*internal market*” did not reach maximum centrality in any national strategy except for Sweden—which strongly focused on the Nordic market, and less on the EU internal market—it became relatively more central in three countries in TP2 as compared to TP1. Based on these results, we can only partly confirm H1.1: whereas the internal market became more central, supply security was already central in TP1 and there was no clear trend of increasing centrality.

“*Limiting costs*” became relatively more central to countries’ strategies between TP1 and TP2, but there was no clear trend of “*technology neutrality*” becoming increasingly central in our sample of countries. We can therefore also only partly confirm H2.1.

Reliance on “market forces” slightly increased in relative centrality between TP1 and TP2, with a simultaneous decline in centrality of “state forces.” Spain poses an exception, however, as its strategy in TP2 does not discuss instrumental logic. As “limiting costs” also became relatively more central, we can confirm H3.1.

The analysis of Fig. 2 indicates variation in the degree of change in policy strategies between TP1 and TP2 (see Sect. 3.1). Sweden, France, and the UK, with change rates between 5 and 7, marked relatively few strategic changes. For Spain, we see no strategic changes in goals, but strong changes in the country’s objectives and instrumental logic (change rate 8). Italy and Germany with change rates between 11 and 13 undertook stronger strategic changes, where central goals and objectives in TP1 did not remain central, and new foci were added in TP2. Italy’s drastic changes are partially explained by its lack of a comprehensive plan to shape EU climate policy in TP1. With its strategy in TP2, Italy moved from solely focusing on the implementation of the Kyoto target to a comprehensive climate and energy policy position. Germany’s strategic shift is noteworthy as the country repositioned itself regarding several design elements, most significantly regarding its instrumental logic moving from a focus on “state forces” in TP1 to solely wanting to rely on “market forces” in TP2. This finding is in line with previous research that has shown that Germany undertook rather drastic changes around 2017, when it had been grappling with an increasing unpopularity of their feed-in tariff schemes, which were deemed very costly (del Río & Mir-Artigues, 2012; Leiren & Reimer, 2018; Markard, 2018; Meckling et al., 2015). The German strategy update in 2017 also supports our assumption that major position shifts are followed by publication of a new strategy document (see “Data and Methods” section). The German strategy update in 2017 also supports our assumption that major position shifts are followed by publication of a new strategy document (see “Data and Methods” section).

For goals, we see important changes in the prioritization of “*limiting costs,*” which became increasingly central in TP2 (change rate 6). We also find strong changes in “*climate and environmental protection*” (change rate 7); however, the change direction varied across countries—gaining priority in some while losing in others.

For objectives, the elements “*internal market*” and “*technology neutrality*” were marked by a higher level of strategic change (both change rate 5), whereas “*energy efficiency*” and “*renewables*” remained relatively stable (change rate 2 and 3) between TP1 and TP2. We observed the common trend of “*internal market*” becoming more prominent, with three countries—Germany, Spain, and Italy—adding the objective “*internal market*” to their strategies. The centrality of “*technology neutrality*” changed slightly across countries, and remained a strategic element in Germany, the UK, France, and Sweden; on the other hand, Italy added “technology neutrality” to its strategic agenda.

Looking at instrumental logic, we find minor strategic change toward “(reliance on) market forces,” but a very high change rate (12) for the element of “(reliance on) state forces,” which decreased strongly in TP2. Directly comparing reliance on “state forces” and “market forces,” we find a common trend toward prioritizing “market forces” as the dominant instrumental logic in TP2 (in four of six countries). In countries where “(reliance on) state forces” was placed centrally in TP1—Germany, the UK, Spain, and France—the element disappeared from their strategies in TP2, now favoring “(reliance on) market forces.” The countries that relied more on market forces in TP1—the UK, Italy, and Sweden—continued to place this element centrally in TP2—although Italy and Sweden also found a role for “state forces.” In summary, between TP1 and TP2, “(reliance on) market forces” either gained in centrality in countries’ strategies or remained a stable influence. Spain represents an outlier in this regard, in its omittance of instrumental logic in TP2. The increased focus on “market forces” as an instrumental logic in TP2 can likely be explained by an increasing maturity of renewable technologies, where they are no longer regarded as requiring market introduction support schemes (Meckling et al., 2017). This change would also explain the increased reliance on the emissions trading scheme in the 2030 climate and energy package.

### Link centrality within climate policy-leading countries’ policy strategies

**Fig. 3:**
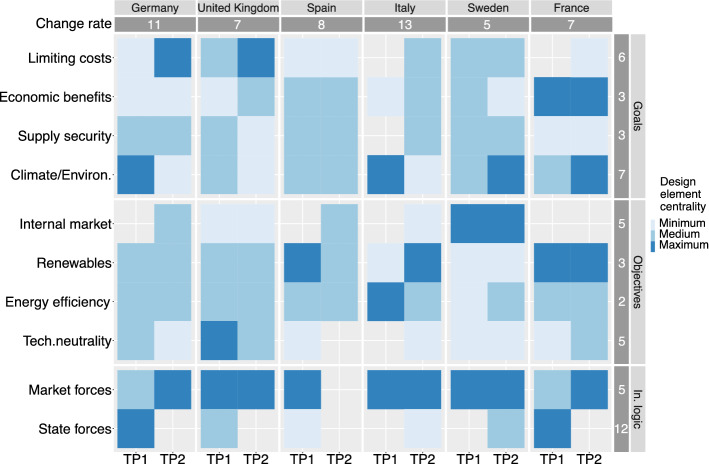
Degree centrality as percent of overall elements mentioned in one design element category split into three groups: minimum < 20%, medium = 20–40%, maximum > 40%. Note: TP1 = the last strategy prior to the adoption of the 2020 climate and energy package; TP2 = the last strategy prior to the adoption of the 2030 climate and energy package (TP2). Empty squares indicate that the respective goal or objective is not part of a country’s strategy

**Fig. 4:**
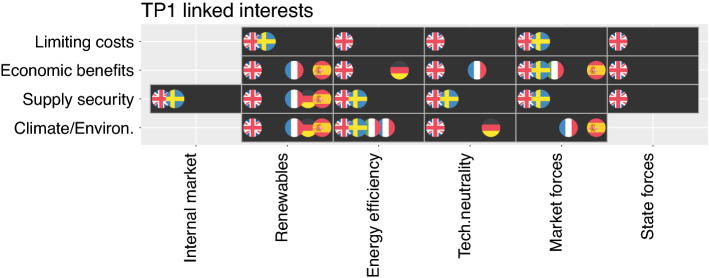
Link centrality in affiliation matrix for goals (y-axis) and objectives/Instrumental logics (x-axis). Linked elements are marked with a flag for the UK, Sweden, Italy, France, Germany, and Spain, respectively. TP1 = the last strategy prior to the adoption of the 2020 climate and energy package. Empty squares indicate that the respective links do not occur in any country’s strategy

Figure 3 (TP1) and Fig. 4 (TP2) show the central links within climate policy-leading countries’ policy strategies in affiliation matrices, with objectives and instrumental logics shown on the X-axis and goals on the Y-axis; the difference between the figures indicates the changes from TP1 to TP2. A flag denotes that a country linked a goal and objective.

**Fig. 5:**
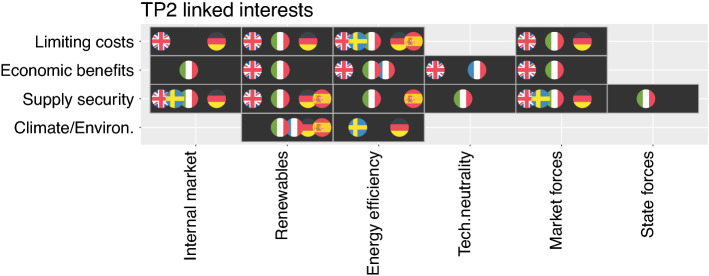
Link centrality in affiliation matrix for goals (y-axis) and objectives/ Instrumental logics (x-axis). Linked elements are marked with a flag for the UK, Sweden, Italy, France, Germany, and Spain, respectively. TP2 = the last strategy prior to the adoption of the 2030 climate and energy package. Empty squares indicate that the respective links are not made within any country’s strategy

Based on our results, we confirm H1.2. We find that most states strategically linked “*supply security*” and “*internal market*” in TP2, while only the UK and Sweden linked these goals in TP1. However, we cannot confirm H2.2. In TP1, only the UK linked the goal of “*limiting costs*” to “*technology neutrality,*” but no country made this connection in TP2. We find weak evidence for H3.2. While reliance on “market forces” is becoming increasingly linked to “limiting costs”—a link now made by three instead of two countries in TP2—we see a parallel shift away from linking “market forces” to economic benefits in TP1 to “supply security” in TP2 (Fig. 5).

For TP1 (Fig. 3), we find four points of ‘close’ alignment, shared by four or more of the climate policy-leading countries, in their argumentative logic. Both “*energy efficiency*” and “*renewables*” were closely linked to “*climate and environmental protection*.” This result echoed the rhetoric of the 2020 climate and energy package: climate protection through both renewables and efficiency. “Renewables” were also closely linked to “supply security.” In addition, “(reliance on) market forces” and “economic benefits” were closely linked.

In TP2 (Fig. 4), “*renewables*” remained closely linked to “*climate and environmental protection,*” but “*energy efficiency*” is mainly linked to the goal of “*limiting costs*”—and less often to “*climate and environmental protection.*” Rather than a central means for climate protection, “*energy efficiency*” became increasingly perceived as a central means to limit costs. This reality does not only indicate a changing argumentative logic around energy efficiency, but also appears to contradict the EU’s “energy efficiency first” principle in the 2030 package. However, considering that limiting costs became an increasingly important goal (see Fig. 2) in TP2, combined with the fact that energy efficiency and limiting costs are discursively closely linked, we find the increasingly cost-focused EU climate policy consistent with an increasing emphasis on energy efficiency.

“*Supply security*” remained closely linked to “*renewables*” in TP2, but was additionally closely linked to the objective of an “*internal market.*” This shift indicates a different argumentative logic for “*supply security*” policy in TP2. In TP1, “*supply security*” was predominantly interpreted as a domestic issue, whereas “*renewables*” were seen as a contributing factor in maintaining domestic “*supply security*” because of their effect on energy import reduction. TP2’s tight link to the “*internal market*” suggests that the system balance problems of renewables gained prominence and that its solution was increasingly seen in expanding the system and integrating in the internal market. Here, changes in domestic strategies indeed preceded the stronger push for the internal market in the 2030 package.

We find a parallel supportive shift in the argumentative logic around the “(reliance on) market forces”: while closely linked to “economic benefits” in TP1, “market forces” are mainly linked to the goal of “supply security” in TP2. This finding is likely related to the increasing focus on the “internal market” as a means for “supply security,” where deregulation and a stronger reliance on “market forces” appears a logical consequence. Such a link would indicate a “diagonal influence” in Fig. 2 linking instrumental logics and objectives.

Countries were generally more closely aligned in their argumentative logic in TP2, although France became an outlier. The country’s weak concern for “*supply security*” in both TPs, likely the result of its strong focus on nuclear power, sets it apart from its neighbors.

## Discussion and conclusion

Our results show that policy changes between the EU’s 2020 and 2030 climate and energy packages were preceded by shifts in the policy strategies of key member states. This claim supports the intergovernmentalist argument that European policy change is more likely to transpire when member states support it as ultimate decision-makers. However, it is also important to acknowledge the continuous interaction between EU and domestic politics and policy. Concretely, all positions post-2009 are affected by what was decided in the 2020 package. Therefore, it is challenging to establish a definitive direction of influence. The movement away from the national toward a much stronger focus on the European level in the 2030 package echoed an increasingly central role of the internal market in national strategies, which more frequently linked supply security to the internal market. A similar shift has been identified as central to CEEC states’ positions (Szulecki et al., 2016), but whereas CCEC countries supported the internal market mainly as a means for joint gas purchases, climate-policy leading countries in the EU link the internal market to the deployment and integration of renewables. In these western, climate-policy leading countries, the internal market is thus also seen as a climate policy, whereas CEEC states tend to view it as a bulwark to safeguard fossil gas supply. The objective of market integration linking to those two opposing goals can be attributed to the Juncker Administration’s purposefully ambiguous design of the “Energy Union,” which includes traditional supply security alongside climate topics (Fischer, 2017b).

Countries increasingly gathered around the internal market and reliance on market forces to optimize supply security and limit transition costs during the run-up to the 2030 package. While the reliance on market forces and the internal market remained a central area of conflict between countries ahead of the 2020 package, greater agreement on that supply security posed a concern, aligned countries’ positions, and helped pave the way for the 2030 package and the policy changes therein.

Changes in domestic objectives in climate-policy leading states in the EU, however, did not precede the increased centrality of technology-neutral decarbonization vis-a-vis technology-specific options, such as renewables and energy efficiency at the EU level. Nevertheless, our results indicate that although climate-policy leading countries did not push for this change, they were also not actively working against it. For the 2030 package, these states moved to support a combination of technology-neutral decarbonization, renewables, and energy efficiency. While the position of climate-policy leading countries proves important for understanding the increasing focus on the internal market in the 2030 climate and energy package, it is not the only change between the two time periods. The changing and increasing influence of CEEC states constitutes another clearly observable change, and, as other research shows (Skjærseth, 2016, 2018), these countries strictly opposed legally-binding renewables targets, thereby supporting our findings for the climate-policy leading countries.

We find that by introducing the idea of a dynamically changing “argumentative logic” to Cashore and Howlett’s taxonomy of design elements (Cashore & Howlett, 2007), we are able to uncover policy changes that would have gone unnoticed in a simple frequency analysis. A main methodological contribution of our study is thus to demonstrate that the hierarchical and static application of the taxonomy falls short of uncovering the ways in which policy requirements for reaching set policy goals become dynamically redefined over time—even if the goals themselves do not change. We believe that by not only disaggregating policy into different elements, but also considering how they are linked to one another, presents a useful avenue for further research. The method we develop and use here to analyze these links provides us with an objective and replicable method for the comparison of argumentative logics that covers a middle ground between frequency analysis and syntax analysis. Because of its replicability, our method lends itself to further automatization, which could lead to further research exploring the ways that automated data collection could facilitate analysis of larger samples of national policies over longer periods of time.

Evaluating our results in the broader context of ongoing system changes, we find that beyond changes in instrumental preferences highlighted in previous studies (Meckling et al., 2017), countries’ goals and objectives have changed significantly. We thus find evidence of countries dynamically redefining their goals relative to the different phases of the energy transition. This finding supports related conclusions in the policy feedback literature (Meckling et al., 2017; Schmidt & Sewerin, 2017). First, the increasingly strong link between energy security and the internal market as a means to maintain system reliability supports the argument that countries perceive a more integrated European market as a tool for grappling with increasing shares of variable renewable power supply. Qualitative analyses of the policy strategies contained in the logbooks of the text coders lend further support to this interpretation. This finding also explains France’s outlier position: in maintaining a strong focus on baseload nuclear power, the country does not face the issue of variable renewable supply. Second, while the combination of increasing cost concerns and technological maturity of renewables does not lead countries to favor broader technology-neutral decarbonization approaches, we find they increasingly favor an instrumental logic that relies on market forces.

Our findings come with two important caveats. First, expected variation occurs in the data coverage of individual countries, e.g., not all countries have formulated a coherent strategic position going into the negotiations of the 2020 and 2030 package, respectively. We are confident that in our case, such variations can be meaningfully interpreted in that the absence of a strategy indicates relatively lower importance of the climate and energy policy field. In the case of Spain, for example, domestic political struggles caused by the Euro crisis of 2010, moved other issues to the top of the political agenda and prevented the country from developing a strategic position after 2011 (Gürtler et al., 2019). In addition, important directional strategic changes would be implemented with the publication of a new strategy, as was the case for Germany in 2017.

Second, in our operationalization of argumentative logic, we focus on links that are made within the same sentence. We recognize this strict criterion may overlook some specific links, but it is the only criterion that can be systematically applied across all countries. Nevertheless, we believe that this approach goes significantly further than solely counting items in frequency analysis, and provides a time-efficient alternative to a far more work-intensive syntax analysis.

We believe that our strategy document analysis lends itself to the analysis of policy design elements beyond mechanisms. These documents go beyond instrumental preferences and day-to-day business and present a strategic narrative for the policy field. Moreover, they lend themselves to comparative studies as countries (at least within the EU) publish long-term climate strategies on a regular basis, thereby providing a comparable format. For future research, “National energy and climate plans” (NECPs), which EU member states are asked to provide in the implementation of the 2030 package, may provide another interesting data source for similar research. However, direct comparisons of NECP analyses with our study may be difficult, as the NECPs are strongly standardized and provide less opportunity for states to highlight their own strategic position, e.g., by giving more space to—for them—particularly strategically-relevant topics.

Our results hold important implications for the implementation of the 2030 climate and energy package. First, renewables triggered by targeted policies remain important in climate-policy leading countries’ strategies, although technology-neutral decarbonization has gained more traction. Despite the rise in technology-neutrality in the 2030 climate and energy package, which is not reflected in the national strategies, we find that member states remain politically committed to renewable energy policy and deployment. Second, the shifted positions of climate-action leaders away from the previous position divide toward a less conflicting situation, will likely support concrete progress toward the “Energy Union” and a truly unified energy market and policy in Europe. With supply security and cost concerns as a driver for the internal market, at least the climate-policy leading countries increasingly align with and commit to the European Commission’s vision of market integration in the “Energy Union.”

## Appendix A

See Tables 2

**Table 2:** List of coded documents

Country	Year	Name
Germany	2007	Eckpunkte für ein integriertes Energie- und Klimaprogramm
Germany	2017	Strom 2030 Langfristige Trends – Aufgaben für die kommenden Jahre
United Kingdom	2007	White Paper on Energy – Meeting the energy challenge”
United Kingdom	2017	The Clean Growth Strategy: leading the way into a low carbon future
Italy	2002	Piano nazionale per la riduzione delle emissioni di gas responsabili dell’effetto serra—2003–2010
Italy	2017	Strategia Energetica Nazionale
Spain	2007	ESTRATEGIA ESPAÑOLA DE CAMBIO CLIMÁTICO Y ENERGÍA LIMPIA HORIZONTE 2007- 2012 -2020
Spain	2011	Plan de Energías Renovables (PER) 2011–2020
France	2009	Plan climat de la France Mise en oeuvre du grenelle Environnement
France	2018	Stratégie Nationale Bas-Carbone
Sweden	2002	Samverkan för en trygg, effektiv och miljövänlig energiförsörjning
Sweden	2018	Energipolitikens inriktning

and

**Table 3:** Overview of renewable energy status and progress in increasing generation from renewables between 2004–2009 and 2009–2019 in the selected countries and the EU average between 2009 and 2019, data source: (Eurostat, 2021)

	Germany	United Kingdom	Spain	Italy	France	Sweden	EU28 (average)*
Renewable energy share 2019	17.3%	12.3%	18.4%	18.2%	17.2%	56.4%	22%
Growth between 2009 and 2019	6.5%	8.9%	5.4%	5.4%	5%	8.9%	6.4%
Growth between 2004** and 2009	4.6%	2.3%	4.6%	6.5%	2.7%	8.8%	3.7%

*The average does not include Malta and Cyprus, which, due to their size, skew the average

**Data before 2004 is not available

3.

## Footnotes

1 The 40% emission reduction target is divided into targets for the Emissions Trading Scheme (ETS) and non-ETS targets, whereas only the latter is legally binding at the member state level.

2 At the time of writing, the EU Commission had just announced its “Fit for 55 package,” which proposes several regulations that aim to reduce emissions by at least 55 percent by 2030, including a 40% target for renewables by 2030 and a 9% reduction in consumption by 2030 compared to baseline projections. On a mechanisms level the EU aims to strengthen Power Purchase Agreements (PPAs).

3 In Fig. 1, the category “limiting cost” combines three distinct types of costs, i.e., “costs of policy” as in the costs to tax payers; “price of electricity” referring to the cost to consumers; and “economic costs” signifying adverse effects on economic competitiveness through stronger regulation.

4 Belgium, Denmark, Estonia, Finland, France, Germany, Italy, Luxembourg, Netherlands, Portugal, Slovenia, Spain, Sweden, United Kingdom.
